# Supplementary material for: Thyroidal and Extrathyroidal Requirements for Iodine and Selenium: A Combined Evolutionary and (Patho)Physiological Approach
Source: Nutrients. 2022 Sep 20;14(19):3886. doi: 10.3390/nu14193886 (PMC9571367; doi:10.3390/nu14193886)
Supplement: Supplementary file 1 [file nutrients-14-03886-s001.zip › nutrients-1919280-supplementary.pdf]

Supplemental Materials for the manuscript:

Thyroidal and extra-thyroidal requirements for iodine and selenium: A combined evolutionary and (patho)physiological approach

Dijck-Brouwer D.A.Janneke, Muskiet Frits A.J., Verheesen Richard H., Schaafsma Gertjan, Schaafsma Anne, Geurts Jan M.W.

Table s1: Recommended amounts (µg/day) of selenium and iodine, as set by the US Institute of Medicine, the FAO-WHO, the European Food Safety Authority, and the Chinese Government.

**Age group**	**US DRI**		**FAO-WHO[1,2,3]**		**EFSA (EU DRI)**		**Chinese DRI[6]**	
	Se	I	Se	I	Se[5]	I[4]	Se	I
0-6 mo	15	110	**6**	**90**			15	85
7-12 mo	20	130	**10**	**90**	15	70	20	115
1-3 y	**20**	**90**	**17**	**90**	15	90		
1-4 y							**25**	**90**
4-6 y			**22**	**90**	20	90	**30**	**90**
4-8 y	**30**	**90**						
7-10 y			**21**		35	90	**40**	**90**
7-12 y				**120**				
10-18 y			**32** (M)<br>**26** (F)					
9-13 y	**40**	**120**						
11-13 y							**55**	**110**
11-14 y					55	120		
≥12				**150**				
>14 y	**55**	**150**						
14-17 y							**60**	**120**
15-17 y					70	130		
>18	**55**		**34** (M)<br>**26** (F)		70	150	**60**	**120**
≥65			**33** (M)<br>**25** (F)					
Pregnancy	**60**	**220**	**28-30**	**250**	70	200	**65**	**220**
Lactation	**70**	**290**	**35-42**	**250**	85	200	**78**	**240**

Figures in bold are RDA (US DRI), PRI (EU) or RNI (Chinese DRI), others are AI.

[1] Untoro et al. Reaching Optimal Iodine Nutrition in Pregnant and Lactating Women and Young Children. WHO statement. Public Health Nutrition 2007;10:1527-1529

[2] FAO/WHO. Selenium. In: *Human vitamin and mineral requirements.* Report of a joint FAO/WHO expert consultation-Bangkok. Rome/FAO 2001:235-250

[3] FAO/WHO. Iodine. In: *Human vitamin and mineral requirements.* Report of a joint FAO/WHO expert consultation-Bangkok. Rome/FAO 2001:181-194

[4] EFSA. Scientific Opinion on Dietary Reference Values for iodine. EFSA J 2014;12:3660

[5] EFSA. Scientific Opinion on Dietary Reference Values for selenium. EFSA J 2014;12(10):3846

[6] Chinese Nutrition Society, Chinese Dietary Reference Intakes, 2013, Beijing, China, cns@cnsoc.org

Table s2: Parameters used by the different institutes (IOM, FAO-WHO, EFSA) to set the iodine recommendation for the different target groups.

Institute	Infants	Children			Adults	Pregnancy	Lactation
		1-3 y	4-8 y	9-13 y			
IOM[1]	(Extra-polated) intake from breastmilk (146 µg/L; 0.78 L/day)	Balance study (4-days, n=7)	Balance study	Extra-polated from adults	Thyroid iodine (^{131}I) accumulation and turnover in euthyroid adults (n=292), and urinary iodine excretion	Adult recommendation + estimated fetal need. Supplementation with 200 µg iodine/day prevented goitre in pregnant women	Adult recommendation + estimated iodine loss in breastmilk
FAO/ WHO[2]	Positive iodine balance	The (inversion) correlation of 24-hour thyroid radioiodine uptake and urinary iodine excretion in 9–13-year-old schoolchildren in rural Mexico (Tovar et al., 1969)			Serum T4, TSH, and urinary iodine excretion	Adult recommendation + estimated fetal need.	Adult recommendation + estimated iodine loss in breastmilk
EFSA[3]	0-6 mo: No thyroid volume/goitre data available <1 year of age	Thyroid volume/prevalence of goitre in relationship to urinary iodine secretion (100 µg/L)				Adult recommendation + fetal, placenta, amniotic fluid, and increased thyroid hormone production	Adult recommendation + loss in breastmilk

[1] IOM: US Institute of Medicine, Food and Nutrition Board [1].
[2] FAO/WHO: Food and Agriculture Organization of the United Nations & World Health Organization [2].
[3] EFSA: European Food Safety Authority [3].

Tolerable upper intake of iodine

Thyroid dysfunction is shown by elevated TSH concentrations. TSH secretion increases when circulating thyroid hormone decreases, and stimulates thyroidal uptake of iodide and the breakdown of Tg in order to release thyroid hormone into the circulation [1]. Using this parameter, the Lowest Observed Effect Level for healthy adults is about 1800 µg/day [1,2,4]. Based on this, and with an uncertainty factor of about 1.5 [1] to 3 [4], the tolerable upper intake (UL) for iodine for all adults has been set at 1000-1100 to 600 µg/day. UL's for children (200-600 µg/day) and adolescents (900 µg/day) are extrapolated from the adult UL based on reference body weights [1] or body surface [4]. For infants, no UL has been established and food and milk should be the only iodine source [1]. The FAO/WHO indicates that an estimated UL for infants is 2 to 3 times the average intake from human milk or 10 times the average iodine content of infant formulas (5 µg/100ml). The FAO/WHO expresses the ULs per kg of body weight: 100 µg (preterm infants), 150 µg (infants 0-6 months, 140 µg (infants 7-12 months), 50 µg (children 1-12 years), 30 µg (adolescents and adults), and 40 µg (during pregnancy and lactation) [2].
In countries with long-standing IDD the intake should not exceed 500 µg/day to avoid the occurrence of hyperthyroidism [4].

Table s3: Parameters used by the different institutes to set the selenium recommendation for the different target groups.

Institute	Infants	Children			Adults	Pregnancy	Lactation
		1-3 y	4-8 y	9-13 y			
IOM[1]	(Extrapolated) intake from breastmilk (18 µg/L; 0.78 L/day)	Estimated average requirement based on the prevention of Keshan disease			Plateauing of plasma glutathione peroxidase	Adult recommendation + estimated fetal need	Adult recommendation + estimated loss in breastmilk
FAO/ WHO[2]	(Extrapolated) intake from breastmilk (18.5 µg/L; 0.78 L/day)	Derived from the adult recommendation based on weight and basal metabolic rate.			Plateauing of plasma glutathione peroxidase	Adult recommendation + estimated need for increased maternal lean mass	Adult recommendation + amount required by the infant
EFSA[3]	(Extrapolated) intake from breastmilk	Derived from the AI for adults by isometric scaling and application of a growth factor.			The relationship between selenium intake and plateauing plasma SEPP1	Adult recommendation	Adult recommendation + estimated loss in breastmilk

See for abbreviations explanation Table s2

Tolerable upper intake of selenium

The tolerable upper intake level is based on the prevention of chronic selenosis taking hair loss and nail brittleness as the most important parameter (most frequently reported) [5]. Chinese studies indicate a LOAEL for selenosis of 900-1000 µg selenium/day [4]. An intake of about 800 µg/day (968 µg/L) in 5 Chinese subjects from a seleniferous area [6], and a chronic intake as high as 724 µg/day (on average 239 µg/day) in US adults, did not result in selenosis [7]. However, at lower intakes reaching 200 µg/L, some small changes have been seen prothrombin time and blood glutathione concentrations of which the clinical relevance is not known [4]. Nevertheless, the 800 µg/day has been used by the IOM to calculated the UL for adults, and with uncertainty factor of 2 the UL for all adults was set at 400 µg selenium per day (which is also the provisionally UL as suggested by the FAO/WHO [2]). The EFSA [8] used 850 µg/day and an uncertainty factor of 3, which resulted in a rounded UL of 300 µg/day for all adults. With regard to pregnant women, there are no reports of teratogenicity or selenosis in the offspring of mothers with high but not toxic intakes of selenium [5]. For young infants, 0-6 months, a human milk with 60 µg/L that did not show known adverse effects was used to calculated the IOM UL (45 µg/day in 780 ml milk; about 7 µg/kg/day) using an uncertainty factor of 1 [9]. The intake of selenium per kg body weight in adults was used to calculate the UL for older infants (60 µg) , children (90 µg for 1-3 y; 150 µg for 4-8 y; 280 µg 9-13 y), and adolescents (400 µg), using reference body weights [5]. Since the uncertainty factor of the EFSA is higher, also the derived ULs for children and adolescents are lower as compared to the IOM Uls: 60 µg/day (1–3 years), 90 µg (4-6 years), 130 µg (7-10 years), 200 µg (11-14 years), and 250 µg (15–17 years) [4].

References Supplemental materials

1. Institute of Medicine Iodine. In *Dietary Reference Intakes for Vitamin A, Vitamin K, Arsenic, Boron, Chromium, Copper, Iodine, Iron, Manganese, Molybdenum, Nickel, Silicon, Vanadium, and Zinc*; The National Academic Press: Washington, DC, 2001; pp. 258–289.

2. FAO/WHO. *Human Vitamin and Mineral Requirements*; FAO Food and Nutrition Division: Rome, 2001;

3. EFSA Scientific Opinion on Dietary Reference Values for iodine. *EFSA J.* **2014**, *12*, 1–57, doi:10.2903/j.efsa.2014.3660.

4. Scientific Committee on Food *Tolerable upper intake levels for vitamins and minerals*; Scientific Committee on Food, Ed.; European Food Safety Authority, 2006; ISBN 9291990140.

5. Institute of Medicine *Dietary Reference Intakes for Vitamin C, Vitamin E, Selenium, and Carotenoids (2000)*; Food and Nutrition board, Ed.; National Academies Press (US): Washington D. C., 2000; ISBN 978-0-309-06949-6.

6. Yang, G.; Zhou, R. Further observations on the human maximum safe dietary selenium intake in a seleniferous area of China. *J Trace Elem Electroytes Heal. Dis* **1994**, *8*, 159–165.

7. Longnecker, M.P.; Taylor, P.R.; Levander, O.A.; Howe, M.; Veillon, C.; McAdam, P.A.; Patterson, K.Y.; Holden, J.M.; Stampfer, M.J.; Morris, J.S.; et al. Selenium in diet, blood, and toenails in relation to human health in a seleniferous area. *Am. J. Clin. Nutr.* **1991**, *53*, 1288–1294, doi:10.1093/ajcn/53.5.1288.

8. EFSA Scientific opinion on the substantiation of a health claim related to vitamin C and increasing non-haem iron absorption pursuant to Article 14 regulation (EC) no 1924/2006. *EFSA J.* **2014**, *12*, 3514.

9. Shearer, T.R.; Hadjimarkos, D.M. Geographic distribution of selenium in human milk. *Arch Env. Heal.* **1975**, *30*, 230–233, doi:10.1080/00039896.1975.10666686.

efe
